# Molecular identification of lactic acid bacteria SR6 strain and evaluation of its activity as an anticancer in T47D cell line

**DOI:** 10.14202/vetworld.2022.1583-1588

**Published:** 2022-06-29

**Authors:** Ida Bagus Ngurah Swacita, I. Wayan Suardana, I. Gusti Ngurah Sudisma, Hevi Wihadmadyatami

**Affiliations:** 1 Department of Preventive Veterinary Medicine, Laboratory of Veterinary Public Health, Faculty of Veterinary Medicine, Udayana University, Denpasar-Bali, Indonesia; 2 Department of Clinical Veterinary Medicine, Faculty of Veterinary Medicine, Udayana University, Denpasar-Bali, Indonesia; 3 Department of Anatomy, Faculty of Veterinary Medicine, Universitas Gadjah Mada, Yogyakarta, Indonesia

**Keywords:** apoptosis, breast cancer, lactic acid bacteria SR6 strain, necrosis

## Abstract

**Background and Aim:**

Breast cancer is the most common type of cancer in women because it attacks the productive age. Preliminary studies showed that lactic acid bacteria (LAB) strain SR6 from the Bali cattle colon has the potential to act as a superior probiotic. It is also assumed that its bacteriocin structure is specific and has a strong relationship with the specificity of the ligand and its biological activity at a receptor. Therefore, this study aims to assess the use of local LAB strains, which produce bacteriocins as anticancer agents, as well as to identify the bacteria as potent producers molecularly.

**Materials and Methods:**

The study was initiated by cultivating LAB SR6 strain from stock isolates on De Man, Rogosa, and Sharpe (Oxoid, CM 0369, England) broth media. It was then confirmed molecularly through analysis of the 16S ribosomal ribonucleic acid gene. Subsequently, its anticancer activity was tested by assessing the cytotoxic activity in T47D cell culture using the 3-(4, 5 dimetiltiazol-2-yl)-2.5-diphenyl tetrazolium bromide (Invitrogen M6494, US) method.

**Results:**

The results showed that the LAB strain SR6 was identified molecularly as *Pediococcus pentosaceus*. Furthermore, it had a toxic effect on T47D cells, which was indicated by the number of deaths after treatment with the extracellular protein of the strain, especially at the 50% total cell volume level.

**Conclusion:**

Based on the toxic effect of the strain on human T47D cells, the LAB SR6 isolate, which was identified as *P. pentosaceus* has the potential to be developed as a good anticancer drug against breast cancer. However, there is a need to carry out an integrated study to fully explore the suitability of bacteriocins as *in vivo* therapeutics against the disease completely.

## Introduction

Breast cancer has a relatively high prevalence and has gained global attention because it affects the productive age in both males and females. Furthermore, it accounts for 16.6% of all cases in Indonesia, with cervical cancer in second place with a proportion of 9.2% [1]. Management of the patients is often carried out through surgery, chemotherapy, hormone, and radiation therapy, but the latest technique is immunotherapy [2]. However, the various treatment methods have side effects, for example, the use of radiation causes mutations in normal cells as well as nausea, baldness, and menopausal syndrome [3].

Probiotics obtained from lactic acid bacteria (LAB) are often used to inactivate the carcinogenic components in the body. Bacteriocins are cationic peptides synthesized on the ribosome and secreted by almost all groups of bacteria, especially the LAB strain. Furthermore, the previous studies [4-6] have reported their therapeutic potential against various types of cancer cells. They have also shown selective cytotoxicity against carcinogenic cells compared to normal variants, which makes them promising candidates for further investigation and clinical trials. Several bacteriocins produced by LAB have been reported to have anticancer effects, such as pyocin, colicin, pediocin, microcin [4], and azurin [5]. Chumchalova and Smarda [6] revealed that Colicin E1 produced by *Escherichia coli* with a molecular size of 57 kDa has similar activity against the MCF-7 breast cancer cell line. The previous studies [7, 8] also reported that Nisin from *Lactococcus latis* and Bovicin HC5 from *Streptococcus bovis* HC5 with molecular weights of 3.5 and 2.4 kDa, respectively, have anticancer effects on the MCF-5 cell line.

Various fractions of LAB, including the whole cell, heat-activated parts, walls, peptidoglycan, and cytoplasmic fractions have anti-proliferative effects on the cancer cell cycle [2, 9]. Furthermore, preliminary studies on the bacteriocin produced by the SR6 strain isolated from Bali cattle colon showed that it has the potential to be a superior probiotic. It is assumed that it has a specific structure that correlates with biological activity. The structure of the bacteriocin-ligand affects its functions as well as in the T47D cell line. There are no studies on the effects of bacteriocin obtained from LAB SR6 on cancer.

Therefore, this study aims to assess the use of bacteriocin produced by local LAB strains as anticancer agents with a toxicity test and to identify the bacteria as the molecular producer.

## Materials and Methods

### Ethical approval

An approval from the Institutional Animal Ethics Committee was not required as no live animals were used.

### Cultivation of LAB strain SR6 isolate

LAB SR6 isolate obtained from the colon of Bali cattle with a high tolerance to the environment was taken from a 30% glycerol stock, which was stored at −20°C. It was thawed at 4°C for 15 min, planted at 27°C in sterile De Man, Rogosa, and Sharpe (MRS) broth media (Oxoid, CM0359, England), and then incubated at 37°C for 24 h. The grown isolate was ready to be used for deoxyribonucleic acid (DNA) extraction and bacteriocin production [10].

### DNA extraction and polymerase chain reaction (PCR) amplification of 16S Ribosomal ribonucleic acid (rRNA) gene

Based on the manufacturer’s procedure with slight modification, the Geneaid Kit and Presto Mini gDNA Bacteria Cat. GBB100 (Genome Life Science, India) was used to extract the DNA of the isolates. Furthermore, the universal primer B27F (5’-AGAGTTTGATCCTGGCTCAG-3’) and U1492R (5’-GGTTACCTTGTTACGACTT-3’) were used to analyze the 16S rRNA [11, 12]. The PCR program was then carried out with a 36 μL total reaction volume, which contains 2 μL DNA template, 25 μL My Taq HS Red Mix, 7 μL distilled water, and 1 μL (20 pmol/μL) of primer 27F and U1492R each. The PCR amplification was performed with an initial DNA denaturation at 94°C for 7 min, followed by 30 cycles, which consisted of denaturation at 94°C for 1min, annealing at 45°C for 45 s, and an extension at 72°C for 1 min. The process was completed with a final extension at 72°C for 5 min, after which 5 μL of the product was analyzed using electrophoresis in 1% agarose [10, 13].

### Sequencing and phylogenetic analysis

The 16S rRNA-sequencing process of the isolate was carried out with a genetic analyzer (ABI Prism 3130 and 3130 × l Genetic Analyzer, Thermo Fisher Scientific, United States) in Singapore through the Institute for sequencing service providers at PT Genetika Science, Jakarta, using similar primers. The sequences were edited to exclude the PCR primer binding sites and corrected with the MEGA X version software (The Pennsylvania State University, USA; https://www.megasoftware.net/) [11, 12]. Furthermore, the full gene sequences were compared automatically using the BLAST program against that of the bacteria deposited in the GenBank of the National Center for Biotechnology Information (NCBI) (www.ncbi.nlm.nih.gov). The neighbor-joining algorithm method was then used to construct the phylogenetic tree [11, 13].

### Preparation of human T47D cancer cell

Monolayer culture of human T47D cell was carried out by culturing 1 mL of the sample under standard conditions at the LPPT laboratory, Gadjah Mada University, in a complete Dulbeccos Modified Eagle Medium (Sigma, D6046). The media were supplemented with 100 IU/mL penicillin, 100 mg/mL Streptomycin, 50 μg fungizon (Fisher Scientific, BW17-745H), and 10% Newborn Calf Serum (Sigma, N4887). Subsequently, the cell was incubated in a humidified atmosphere containing 5% carbon dioxide (CO_2_) at 37°C [12, 14].

### Toxicity assay

A total of 50 μL T47D cells were implanted into 96 well microplates (Merck) and then incubated for 24 h at 5% CO_2_ to obtain confluent growth with a density of 5 × 10^4^ cells/well. The media were then replaced with new variant, and 50 μL of crude toxin with serial dilution was added. Subsequently, the toxins were removed and the monolayer cells were washed two times after incubation for 15 min at room temperature (27°C). 100 µL of the complete growth medium was added to the cells before they were incubated for 24 h at 37°C, and 5% CO_2_. The samples were then washed with phosphate buffer saline solution pH 7.4. 10 μL of 0.5% 3-(4, 5 dimetiltiazol-2-yl)-2.5-diphenyl tetrazolium bromide (MTT) reagent and 100 μL of culture media were added to each well. The cells were incubated at 37°C for 4–6 h in 5% CO_2_ to form formazan, and the reaction was stopped by adding 100 μL of MTT reagent stopper. They were incubated overnight, and then analyzed by an enzyme-linked immunoassay reader at l 550 nm [12, 14].

## Results

### Molecular Identification of LAB SR6

**Figure-1:**
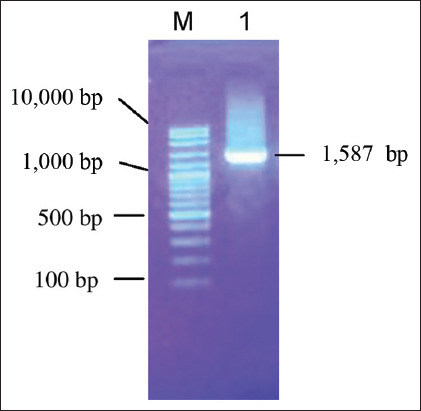
Amplification of the 16S rRNA gene of the LAB SR6 gene with universal primers 27(F) and 1492(R) on 1% agarose. M: Markers; 1: isolate SR6. rRNA=Ribosomal ribonucleic acid, LAB=Lactic acid bacteria.

The LAB SR6 isolate stored in a freezer at −20°C was cultivated on MRS broth media. The identification process started with total DNA extraction, followed by PCR on the 16S rRNA gene using universal primers B27F and U1492R. Figure-1 shows the product obtained on 1% agarose.

Figure-1 revealed that the 16S rRNA gene of the LAB SR6 isolate was successfully amplified, which was indicated by the 1587 bp length obtained. The PCR product was then sequenced to determine the composition of their constituent nucleotides. Furthermore, Table-1 shows the nucleotide alignment results between the isolate and others in the GenBank of the NCBI, such as *Pediococcus pentosaceus* strains (AB682664), *Pediococcus siamensis* (AB258357), *Lactobacillus rhamnosus* (D16552), *Enterococcus faecalis* (JX2421472), *Aerococcus viridans* (LC487893), *Aerococcus viridans* (AB680271), *Lactococcus lactis* (AB100796), *L. lactis* (LT853603), *Leuconostoc lactis* (AJ970316), *Leuconostoc mesenteroides* (AB596940), *Oenococcus Kitahara et* (AB221475), *Oenococcus oeni* (AB596939), and *Bifidobacterium breve* (NR040783).

**Table 1:** Nucleotide statistics of the 16S ribosomal ribonucleic acid gene of the SR6 isolates with the several strains of *Aeromonas*, *Bifidobacterium*, *Enterococcus*, *Lactobacillus*, *Lactococcus*, *Leuconostoc*, *Oenococcus*, and *Pediococcus* registered in the GeneBank.

Strains	T (U)	C	A	G	Total
Isolate SR6	22.1	22.1	26.7	29.0	1436
*A. viridans* (LC487893)	21.8	21.9	26.7	29.6	1309
*A. viridans* (AB680271)	21.4	22.5	26.5	29.7	1469
*Bifidobacterium breve* (NR040783)	19.8	24.9	20.9	34.3	1520
*Enterococcus faecalis* (JX2421472)	21.4	23.7	24.4	30.5	987
*Lactobacillus rhamnosus* (D16552)	21.9	22.2	25.9	30.0	1515
*L. lactis* (AB100796)	21.6	21.3	27.0	30.1	1499
*L. lactis* (LT853603)	21.5	21.7	26.7	30.1	1462
*Leuconostoc lactis* (AJ970316)	21.2	22.0	26.9	29.9	1502
*Leuconostoc mesenteroides* (AB596940)	21.7	21.9	26.7	29.7	1473
*Oenococcus kitaharae* (AB221475)	23.0	22.0	26.1	29.0	1493
*Oenococcus oeni* (AB596939)	23.0	21.5	26.8	28.7	1486
*Pediococcus siamensis* (AB258357)	22.0	22.4	25.9	29.6	1488
*Pediococcus pentosaceus* (AB682664)	22.0	21.9	26.8	29.3	1501

*A. viridans=Aerococcus viridans*, *L. lactis*=*Lactococcus lactis*

**Figure-2:**
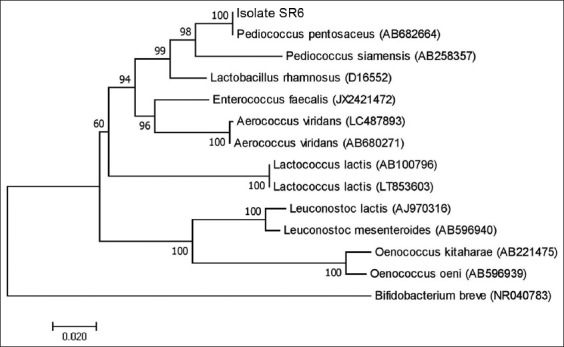
Phylogram constructed using the Neighbor-Joining algorithm from the nucleotides of the 16S rRNA gene. Figures in the phylogenetic branch show the bootstrap value (%). rRNA=Ribosomal ribonucleic acid.

Table-1 shows that the 16S rRNA gene of the isolate has a similar composition to others, namely, 22.1% thymine (Uracil), 22.1% cytosine (C), 26.7% adenine (A), and 29% guanine (G) of the total 1436 nucleotides. The composition was then used to construct a phylogenetic tree, which determined its group and that of other strains. Figure-2 shows the tree constructed with the Neighbor-Joining algorithm, while the genetic distance is presented in Table-2.

**Table 2:** The genetic distance of the nucleotide arrangement of the 16S ribosomal ribonucleic acid gene of SR6 isolates with several isolates registered in the gene bank using the maximum composite likelihood method in 1000 bootstrap times.

Strains	Isolate SR6	*A. viridans* (LC487893)	*A. viridans* (AB680271)	*B. breve* (NR040783)	*E. faecalis* (JX2421472)	*L. rhamnosus* (D16552)	*L. lactis* (AB100796)
Isolate SR6		0.017	0.017	0.049	0.016	0.010	0.022
*A. viridans* (LC487893)	0.094		0.001	0.047	0.012	0.013	0.023
*A. viridans* (AB680271)	0.093	0.001		0.047	0.012	0.014	0.023
*B. breve* (NR040783)	0.278	0.274	0.273		0.046	0.044	0.051
*E. faecalis* (JX2421472)	0.085	0.061	0.059	0.264		0.013	0.022
L*. rhamnosus* (D16552)	0.043	0.072	0.070	0.253	0.067		0.023
*L. lactis* (AB100796)	0.120	0.128	0.127	0.287	0.119	0.128	
*L. lactis* (LT853603)	0.120	0.128	0.127	0.287	0.119	0.128	0.000
*Leuconostoc lactis* (AJ970316)	0.144	0.144	0.142	0.293	0.127	0.139	0.170
*L. mesenteroides* (AB596940)	0.139	0.142	0.140	0.294	0.126	0.134	0.162
*O. kitaharae* (AB221475)	0.180	0.190	0.189	0.327	0.176	0.175	0.205
*O. oeni* (AB596939)	0.174	0.185	0.184	0.341	0.174	0.173	0.194
*P. siamensis* (AB258357)	0.057	0.117	0.115	0.291	0.097	0.070	0.154
*Pediococcus pentosaceus* (AB682664)	0.000	0.094	0.093	0.278	0.085	0.043	0.120

**Strains**	***L. lactis* (LT853603)**	***Leuconostoc lactis* (AJ970316)**	***L. mesenteroides* (AB596940)**	***O. kitaharae* (AB221475)**	***O. oeni* (AB596939)**	***P. siamensis* (AB258357)**	***P. pentosaceus* (AB682664)**

Isolate SR6	0.022	0.026	0.025	0.032	0.031	0.012	0.000
*A. viridans* (LC487893)	0.023	0.025	0.025	0.033	0.032	0.021	0.017
*A. viridans* (AB680271)	0.023	0.025	0.025	0.033	0.032	0.021	0.017
*B. breve* (NR040783)	0.051	0.051	0.051	0.058	0.061	0.051	0.049
*E. faecalis* (JX2421472)	0.022	0.023	0.023	0.031	0.031	0.019	0.016
*L. rhamnosus* (D16552)	0.023	0.025	0.024	0.031	0.031	0.014	0.010
*L. lactis* (AB100796)	0.000	0.030	0.029	0.036	0.034	0.028	0.022
*L. lactis* (LT853603)		0.030	0.029	0.036	0.034	0.028	0.022
*Leuconostoc lactis* (AJ970316)	0.170		0.005	0.022	0.023	0.031	0.026
*L. mesenteroides* (AB596940)	0.162	0.016		0.022	0.023	0.031	0.025
*O. kitaharae* (AB221475)	0.205	0.117	0.122		0.006	0.036	0.032
*O. oeni* (AB596939)	0.194	0.125	0.126	0.021		0.035	0.031
*P. siamensis* (AB258357)	0.154	0.173	0.172	0.206	0.202		0.012
*Pediococcus pentosaceus* (AB682664)	0.120	0.144	0.139	0.180	0.174	0.057	

*A. viridans=Aerococcus viridans, B. breve=Bifidobacterium breve, E. faecalis=Enterococcus faecalis, L. rhamnosus=Lactobacillus rhamnosus, O. kitaharae=Oenococcus kitaharae, L. lactis=Lactococcus lactis, L. mesenteroides=Leuconostoc mesenteroides, O. oeni=Oenococcus oeni, P. siamensis=Pediococcus siamensis,*

*P. pentosaceus=Pediococcus pentosaceus*

The value presented in blue number (top diagonal) is the estimated standard error value obtained through the bootstrap procedure of 1000 repetitions

**Figure-3:**
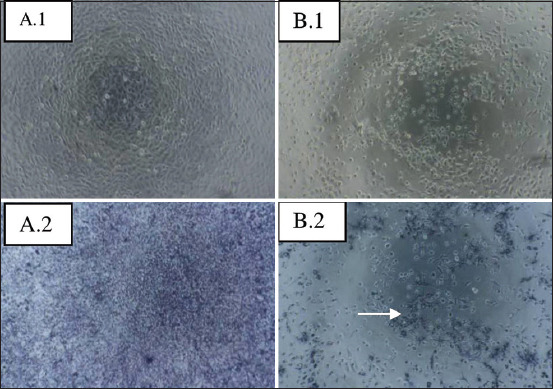
Results of T47D cell hybridization with the extracellular fluid of *Pediococcus pentosaceus* SR6 STRAIN. Photos were taken with a phase-contrast microscope (Olympus, type INT-2/605029) with a magnification of 10 × 40. A1 and B1: Cells before MTT staining were control cells (without treatment), and cells treated with protein supernatant 50%. A2 and B2: Cells after MTT stained 24 h after treatment. The arrow indicates the dead.

Philogram (Figure-2) shows that the LAB SR6 isolate is identical to *P. pentosaceus* (AB682664) with a bootstrap value of 100%. Furthermore, its clade variant also has fewer similarities to *P. siamensis* (AB258357) with 98% bootstrap. Based on the results, it was confirmed to be *P. pentosaceus* based on its molecular composition. The phylogram analysis in Figure-3 was further clarified by analyzing the genetic distance between the isolates compared, as shown in Table-2.

### Anticancer cytotoxicity test of *P. pentosaceus* SR6 strain on T47D cells

The anticancer activity test of the *P. pentosaceus* SR6 strain on T47D cancer cells started with a cytotoxicity test in the form of extracellular fluid treatment for 24 h. Figure-3 shows the cell growth before and after the administration.

Figure-3 shows the presence of several dead cells after they were treated with the extracellular fluid of *P. pentosaceus* SR6 stain. Meanwhile, after staining with MTT as a control, only a few cells were still alive. These findings indicate that the extracellular fluid or supernatant of the strain has a toxic effect on T47D cells.

## Discussion

The molecular analysis identified the LAB SR6 isolate as *P. pentosaceus* and grouped it into one clade as the bacteria (AB682664) with a bootstrap value of 100%. This finding is consistent with Janda and Abbott [15] on the rules for microbial identification based on the 16S rRNA sequencing. Furthermore, the criteria for identifying similarities between species include a minimum sequence similitude of >99% and an ideal value of >99.5%. The results obtained differ from those of previous studies that identified LAB strains from Bali cattle, such as LAB SR9 as *L. lactis* spp. lactis 1 [16], LAB SR2 as *L. lactis* [10], and LAB 9A as S*. bovis* [11].

*P. pentosaceus* is a LAB that has played an increasing role in recent years. Furthermore, it has been isolated from fermented food, aquatic products, raw animal, plant products, and feces. Some strains have also been reported to be associated with the human gastrointestinal tract [17]. A previous study revealed that pentocins L and S produced by *P. pentosaceus* with molecular weights of 27 and 25 kDa, respectively, have broad inhibition spectra and are thermostable. They can also inhibit the growth of tested spore-forming G (+) and G (−) strains as well as the germination of *Bacillus subtilis* ATCC 10225, *B. subtilis* ATCC 10254, and *Bacillus cereus* ATCC 11778 spores [18]. Another study stated that pediocin K23-2 produced from *P. pentosaceus* K23-2 in Kimchi with 5 kDa MW has the potential to be used in the food and feed industries as natural bio preservatives. It can also be used as a probiotic for humans and livestock [19]. Ayyash *et al*. [20] revealed that *P. pentosaceus* M41 can produce EPS-M421, which has an average molecular weight of 682.07 kDa, as well as 77.5% and 46.4% antitumor inhibition against Caco-2 and MCF-7 cells, respectively. Several studies have explored the anticancer effect of bacteriocin in breast cancer. Furthermore, Chumchalova and Smarda [6] reported that Colicin E1 produced by *E. coli* with 57 kDa size has toxic activity against the MCF-7 cell line. Other studies also revealed that Nisin from *L. latis* and Bovicin HC5 from *S. bovis* HC5 with molecular weights of 3.5 and 2.4 kDa, respectively, have similar effects on the MCF-5 cell line [7, 8]. These findings are consistent with this study, which observed that the LAB SR6 strain isolate is toxic to the T47D cells. This was indicated by the number of death that occurred after the samples were treated its extracellular protein, especially at 50% total cell volume level. This toxicity against T47D cells was predicted by the pediocin produced by LAB SR6 based on the molecular identification of the isolate as a *P. pentosaceus*.

Several studies have reported the therapeutic potential of bacteriocins produced by LAB on various cell lines. Furthermore, some of the products have selective cytotoxicity toward cancer cells compared to normal cells without the exception of pediocin produced by *Pediococcus* spp. [4]. Pediocin CP2 from *Pediococcus acidilactici* CP2 MTCC5101 as well as its recombinant version was tested for their cytotoxic effects against various human cancer cell lines, such as HepG2, HeLa, and MCF-7. The MCF-7 cell lines retained percentage viabilities of 2.13 and 10.74, while HepG2 retained 5.52 and 1.23% [21].

LABs’ mechanism of action in cancer cells is through the extrinsic and intrinsic pathways of apoptosis, where they serve as the activator. The extrinsic pathway triggers the Fas/tumor necrosis factor receptors or other factors to induce the caspase-related pathways. Meanwhile, the intrinsic pathway requires mitochondrial localization as well as the activation of Bax and Bak. LAB can enhance the apoptosis induction capacity of 5-fluorouracil and activate autophagic cell death. The autophagy process is directly facilitated by the induction of Beclin-1 and GRP78, as well as indirectly through the induction of Bcl-2 and Bak. The bacteria can also prevent cancer by downregulating the nuclear factor-kappaB-dependent gene products, which regulate cell proliferation (Cox-2, cyclin D1) and survival (Bcl-2, Bcl-xL) [3]. Based on these actions, the pediocin produced by LAB SR6 strain can facilitate the entrance of T47D cell lines into the apoptosis cycle.

## Conclusion

The result showed that bacteriocin produced by LAB SR6 strain has toxic effects on the T47D cell lines. Furthermore, the isolate was identified as *P. pentosaceus* based on its molecular composition. These findings can be used to fully explore the suitability of bacteriocin as *in vivo* anticancer therapeutics against breast cancer in the future. However, there is a need to carry out an integrated study to fully explore the suitability of bacteriocins as *in vivo* therapeutics against the disease.

## Authors’ Contributions

IBNS and IWS: Conceived, designed, and supervised the study. IGNS and HW: Analyzed the data and edited the manuscript. IBNS: Collected the isolates and performed laboratory activities. All authors have read and approved the final manuscript.
